# Level measurement, regional variations, and the dynamic evolution of active aging in China—analysis based on CHARLS tracking data

**DOI:** 10.3389/fpubh.2024.1504907

**Published:** 2025-01-03

**Authors:** Jiru Guo, Xiaoli Zhang, Longyin Chen, Hong Yang

**Affiliations:** ^1^School of Economics and Management, Shanghai University of Sport, Shanghai, China; ^2^School of Physical Education, Xi’an Physical Education University, Xi’an, China; ^3^School of Physical Education and Sport Science, Fujian Normal University, Fuzhou, China; ^4^School of Art, Xi’an Physical Education University, Xi’an, China; ^5^School of Sports Economics and Management, Xi’an Physical Education University, Xi’an, China

**Keywords:** active aging, level measurement, regional variations, dynamic evolution, China Health and Retirement Longitudinal Study

## Abstract

**Introduction:**

Given the world’s largest and increasingly serious aging population, China has elevated “positively responding to aging of population” to a national strategy. Exploring the current state and evolutionary trends of active aging over the past decade is a fundamental prerequisite and the primary task for implementing this strategy.

**Methods:**

Based on data from the China Health and Retirement Longitudinal Study (2011-2018), this study primarily employs methods such as the entropy method, Gini coefficient, Moran index, and Kernel density estimation to analyze the development level, regional differences, and dynamic evolution of active aging in China.

**Results:**

(1) From a general point of view, the overall level of active aging in China has not been high in the past decade, but has shown a rising trend year by year. Older Chinese people exhibit high levels of physical and mental health, but social participation and economic status remain areas of weakness in active aging. (2) Inter-regional differences are the main source of the overall differences in the level of active aging in China. (3) There is a spatial clustering of the active aging level in China, along with a neighborhood effect. (4) The bifurcation phenomenon of active aging in China has intensified over time. While the eastern region exhibits uneven development, the central and western regions have generally had more balanced growth.

**Discussion:**

To improve the level of active aging among older adult individuals in China, policymakers should continuously optimize policies and pay more attention to the economic status and social participation of the older adult. Local governments should not only fully leverage their regional advantages but also interact with other regions to achieve cross-regional joint development.

## 1 Introduction

Population aging has been and will continue to be one of the most significant processes and factors of the 21st century (or at least the early decades of the 22nd century) (1). This demographic shift brings challenges such as labor shortages, increased healthcare expenditures, and greater burdens on social security systems (2). Insufficient attention to aging issues can lead to social tensions, intergenerational conflicts, and political and social instability. Currently, aging is a global phenomenon (3). As of now, 22 countries and regions have at least 20% of their populations aged 65 or older (4), with the United Nations predicting this number will rise to 59 by 2030 (5). China holds the largest older adult population globally (6), with 216.76 million people aged 65 and older in 2023, a figure set to grow to 260 million by 2030. Focusing and solving aging issues in China can contribute significantly to maintaining stability within the country and mitigating global population aging.

The concept of “active aging” emerged in gerontology in the latter decades of the twentieth century, as the world sought solutions to population aging. Researchers advocate that active aging is an umbrella concept (7), morally sound and economically valuable (8), which can positively impact macro, meso, and micro levels, establishing it as a new paradigm in gerontology (9). The Chinese government has recognized the importance of responding proactively to population aging as a key national strategy. Nevertheless, achieving this strategic goal is challenging. Approximately 190 million older adult people in China suffer from chronic diseases (10), nearly a third have depressive tendencies (11), and the pension incomes for older adult people aged 60 and above in rural areas generally fall below the national poverty line (12). Given these challenges, it is crucial for China to adopt positive means and strategies to address aging. The fundamental prerequisite and primary task in this endeavor is to clarify the current state and evolution trends of active aging in the country.

Current research on active aging is still in its developmental stage and faces several challenges: in terms of doctrine, the connotation of active aging lacks detailed descriptions of construction paths and development strategies, leaving much room for exploration. In terms of empirical evidence, existing studies are limited to specific geographic areas or single years, lacking large-scale national measurements and descriptions of spatial and temporal dynamics. This limitation hampers the ability to present a comprehensive view of the overall development of active aging across the country, regional variations, temporal evolution, and spatial relationships. Given these gaps, this study aims to answer the following questions: What are the characteristics of active aging? What is the development status of active aging in China over the past decade? Are there spatial correlations? What kind of dynamic trends have been observed? Clarifying these questions will help to understand the development of active aging in China, identify its shortcomings, and provide a basis for formulating effective intervention strategies for an aging society. Such insights will aid in promoting the decision-making and strategic deployment of China’s active aging strategy.

## 2 Literature review

### 2.1 Connotation of active aging

Since the 1940s and 1950s, “active aging” has gradually attracted attention from various stakeholders. To this day, scholars still argue that “active aging” lacks a clear conceptual definition (13, 14), primarily because its dimensions are not uniform. Some interpretations focus on a single dimension. For instance, certain industrialized countries center their active aging policies on economic factors (15). Similarly, U.S. policymaking organizations often measure only “participation in moderate-intensity sports” (14). However, the multidimensional nature of “active aging” is more widely recognized compared to focusing on a single dimension. For instance, Hsu et al. proposed that employment, community engagement, independent living, health and security, positive aging ability, and a supportive environment represent the essence of active aging (16). Hui et al. incorporated physical vigor, life satisfaction, family support, and active participation into the concept of active aging (17). Rascado et al. contended that active aging comprises two primary components: health and participation (18). Despite their varying focal points, these studies are all aligned with the three-dimensional framework of “health, participation, and security” proposed by the World Health Organization in its “Active Aging: A Policy Framework.”

This study does not adopt the “three-dimensional framework” proposed by the WHO as the definition of active aging in China. This decision is based on two primary considerations. First, as a country with an exceptionally large and rapidly aging population, China requires a clear strategy for developing pathways and methods for building an active aging society, instead of relying solely on the broad framework proposed by WHO. Secondly, China’s unique context of “aging before affluence” and “aging before readiness” must be considered, rather than limiting the perspective to the globalized view provided by the three-dimensional framework. These unique factors should be explicitly addressed in national policy practices and theoretical research. For example, the “Opinions on Further Strengthening the Cultural Construction for the Elderly” emphasizes optimizing the cultural environment for the older adult and enriching cultural products for them. The “Opinions on Further Strengthening the Preferential Treatment for the Elderly” highlights the need to provide various forms of economic subsidies, preferential treatments, and convenient services for the older adult. The “Opinions of the General Office of the State Council on Formulating and Implementing Elderly Care Service Projects” focuses on tasks such as establishing subsidy systems for economically disadvantaged, older adult people of advanced age and those with disabilities, and strengthening the modification of community and household facilities to be suitable for the older adult. The “Opinions on Strengthening the Work on Aging in the New Era” proposes several initiatives such as improving the multi-level pension security system, enhancing health services and management for the older adult, promoting social participation of the older adult, building a social network for the older adult, creating a livable environment, fostering the silver-hair economy, and enhancing relevant support policies. Furthermore, the “14th Five-Year Plan for Healthy Aging” aims to improve the preventive healthcare service system focusing on both physical and mental health. Lastly, the “Opinions on Promoting the Construction of the Basic Elderly Service System” suggests formulating and implementing a list of basic older adult care services (19–24), and so on.

Additionally, scholars like Fang and Sim and Lee and Tan have suggested specific measures, including organizing classroom activities for the older adult, creating a “third space” in communities for social interaction, and subsidizing physical activity programs as well as parks equipped with amenities (25, 26). These elements respond to and refine the three-dimensional framework. By combining practical requirements at the national governance level and theoretical strategies at the academic research level, it becomes clear that the connotation of active aging should encompass both the ultimate goals and detailed content specified in the “three-dimensional framework.” In this paper, the connotation of active aging in China is summarized as follows: multidimensional physical health, sustainable mental health, diversified social participation, three-dimensional economic status, a suitable physical environment, and full social security coverage.

### 2.2 Measurement tool for active aging

The World Health Organization has proposed the need to develop tools for measuring and monitoring active aging, prompting scholars globally in relevant fields to embark on exploration. For example, Thanakwang and Soonthorndhada compiled the Thai scale for active aging (AAI-Thai), containing three dimensions, which are “health” “community participation” and “security.” And they further supplemented and refined it in 2014, proposing that the scale for active aging should include seven aspects: being self-reliant, being actively engaged with society, developing spiritual wisdom, building up financial security, maintaining a healthy lifestyle, engaging in active learning and strengthening family ties to ensure care in later life (27, 28). Zaidi et al. developed the EU scale for active aging (AAI-EU), which contains individual factors and environmental factors. And this scale has been widely used in subsequent research (29). Buys and Miller designed the AAQ-AUS scale (Australia), which includes work, study, social interaction, emotions, spirituality, health, family environment, and life events (30). Zasimova and Sheluntcova first developed a Russian scale for active aging, with AAI-RUS containing health, social engagement, and security as its three dimensions (31). Rantanen et al. determined the UJACAS scale (Finland) based on what individuals “want to do,” “can do,” “believe is meaningful to do,” and “actually do,” including indicators such as participation in study or community activities, outdoor recreation, maintenance of physical exercise, conscious exercise of thinking and memory, and social engagement (32).

Chinese scholars have also continuously contributed their efforts. For example, in 2012, Hu constructed the Chinese scale for active aging (AAI-CHN) from the perspectives of individuals and the social environment (33). However, the content of active aging in China has undergone many changes over the past decade, making the scale’s content less timely. In contrast, Xie’s study in 2019 was more comprehensive. However, his study proposed “establishing a model of active aging under Chinese culture” (34). What’s more, the study proposed that the concept of “Chinese culture” is too broad, potentially leading to content redundancy in the model. Zhou et al. enriched the indicators of active aging by incorporating economic security, policy support, materials, and facilities, but lacked validation of reliability and validity or only partially presented them (35). It is obvious that domestic Chinese scholars are at the initial stage of constructing indicators and developing scales for active aging currently.

### 2.3 Measurement level of active aging

To address the current status of the level of active aging in China, some scholars have conducted preliminary studies. For example, Pham et al. and Um et al. calculated the index of active aging in China using the Vietnam and EU scales, respectively (36, 37); Tian et al. measured the level of active aging among rural disabled older adults in Henan Province using the Barthel index and a scale adapted from Thailand (38); Liu et al. used the same scale to survey 253 older adults in the community of Yanji City, and the results showed that the total score of positive aging among older adults was 99.22 ± 13.69, which was at a medium level, and further analyzed the influence of demographic information on it (39); Bian and Wang, on the other hand, based on the EU scale, used comprehensive national data (China Health and Retirement Longitudinal Study 2011–2018, China General Social Survey), calculate the national and four major regions active aging index by entropy value method, and further analyze its coordination level (40).

**Figure 1:**
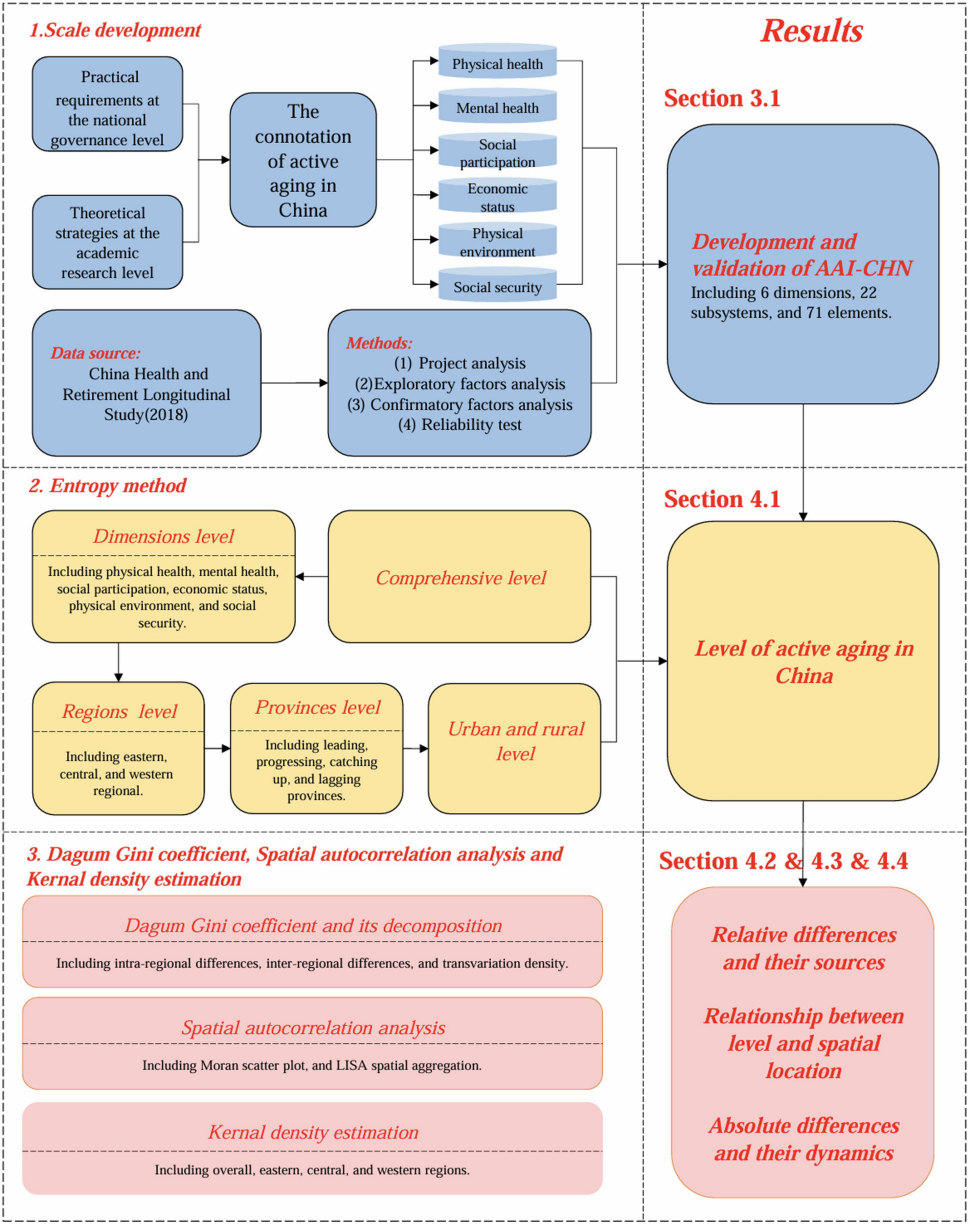
Research framework.

In summary, most existing literature relies on foreign indicator systems, employing small sample data to measure the level of active aging in single regions. These studies are often limited to cross-sectional analyses, with few examining national, dynamic, and regional perspectives. This study aims to address these limitations through the following three aspects: first, construct a multidimensional evaluation scale based on the connotation of active aging in China. Second, measure the current status of active aging in China over the past ten years using the entropy value method. The data sources include the China Health and Retirement Longitudinal Study for the years 2011, 2013, 2015, and 2018. The analysis covers overall, dimensional, regional (eastern, central, and western parts of China), provincial levels and urban and rural level. Third, analyze the relative differences in active aging levels in China and their sources using the Gini coefficient and its decomposition. Additionally, explore the relationship between active aging levels and spatial geography with the Moran index, and present the absolute differences and dynamics over the past decade using Kernel density estimation (see Figure 1).

## 3 Materials and methods

### 3.1 Constructing of active aging scale

**Figure 2:**
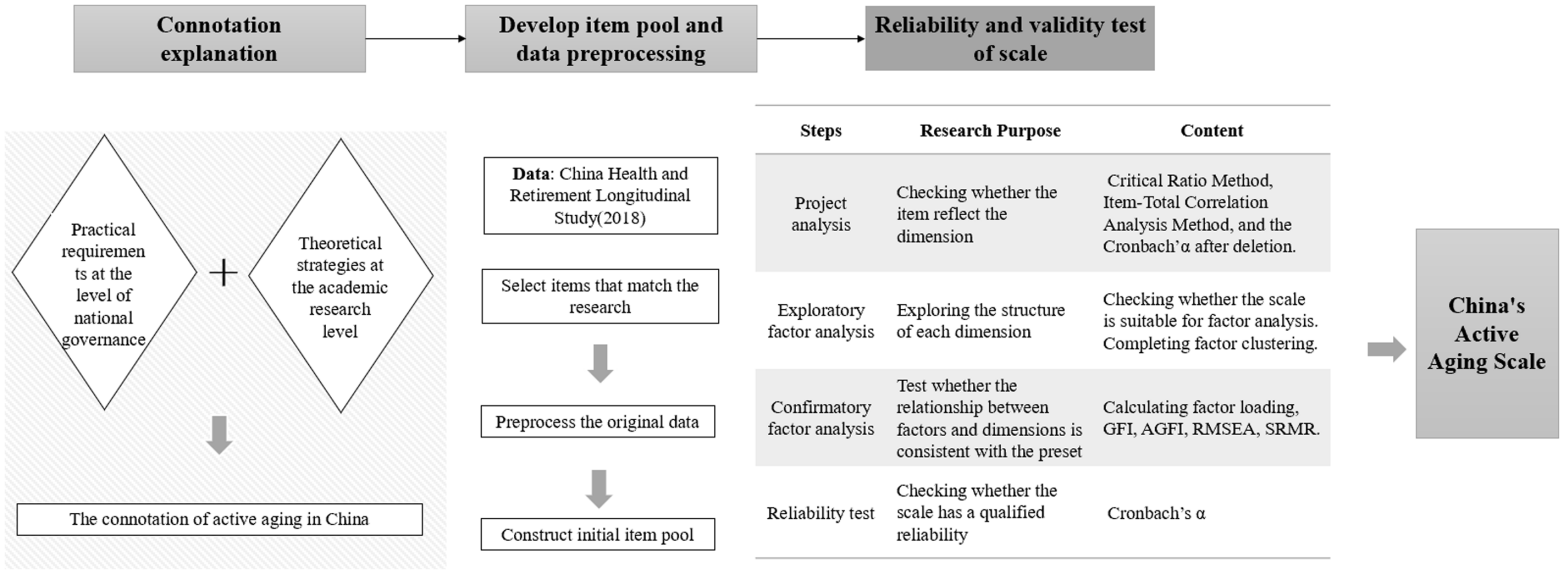
Flowchart for the development of the Active Aging Scale in China.

This study draws on the research ideas of Sin et al., Rantanen et al., and Ayoun and Schmitz, starting from the conceptual definition and connotation analysis (32, 41, 42). After gaining a clear understanding of the concept and connotation of active aging, following the research process outlined in DeVellis and Thorpe’ *Scale Development: Theory and Applications* (1991) and relevant academic papers (43, 44), and considering principles such as scientificity, comprehensiveness, and data availability, this study utilizes data from the China Health and Retirement Longitudinal Study. Through a series of rigorous steps including “develop item pool → data preprocessing →project analysis → exploratory factor analysis → confirmatory factor analysis → reliability test” (as shown in Figure 2), a stable and reliable China’s Active Aging Scale (AAI-CHN) has been developed. The scale includes dimensions such as physical health, mental health, social participation, economic status, physical environment, and social security (as shown in Table 1). The Cronbach’s *α* is 0.867 (≥0.60), GFI = 0.954 (≥0.95), AGFI = 0.940 (≥0.90), RMSEA = 0.050 (≤0.05), and SRMR = 0.047 (≤0.05).

**Table 1:** Active Aging Scale in China.

Dimensions (×6)	Subsystem (×22)	Comprehensive weight	Dimension weight	Element (×71)	Comprehensive weight	Dimension weight
Physical health	Physical function	0.0472	0.2348	A1 Hypermetropia	0.0140	0.0487
				A2 Myopia	0.0143	0.0497
				A3 Hearing	0.0141	0.0491
	Abilities of daily life activities	0.0518	0.2578	A4 Dressing	0.0167	0.0582
				A5 Bathing	0.0167	0.0580
				A6 Eating	0.0168	0.0584
				A7 Getting into or out of bed	0.0167	0.0582
				A8 Making stool	0.0166	0.0578
				A9 Controlling urination and defecation	0.0167	0.0582
	Instrumental self-care ability	0.0515	0.2661	A10 Doing household chores	0.0164	0.0572
				A11 Cooking	0.0164	0.0572
				A12 Shopping	0.0165	0.0574
				A13 Managing money	0.0164	0.0571
				A14 Taking medicine	0.0167	0.0581
	Body behavioral function	0.0505	0.2513	A15 Jogging about 1 km	0.0138	0.0482
				A16 Getting up from a chair after sitting for a long period	0.0166	0.0579
				A17 Climbing stairs	0.0156	0.0545
				A18 Stooping, kneeling, or crouching	0.0161	0.0560
Mental health	Somatic symptom	0.0512	0.2496	B1 I was bothered by things that do not usually bother me.	0.0161	0.0792
				B2 I had trouble keeping my mind on what I was doing.	0.0161	0.0791
				B3 I feel depressed.	0.0161	0.0789
				B4 I felt everything I did was an effort.	0.0160	0.0786
	Positive mood	0.0506	0.2468	B5 I felt hopeful about the future.	0.0155	0.0762
				B6 I was happy.	0.0159	0.0781
	Depressive mood	0.0519	0.2534	B7 I feel fearful.	0.0166	0.0814
				B8 I feel lonely.	0.0164	0.0806
				B9 I could not get “going.”	0.0166	0.0813
	Satisfaction	0.0513	0.2502	B10 Life satisfaction	0.0164	0.0805
				B11 Health satisfaction	0.0133	0.0654
				B12 Marital satisfaction	0.0141	0.0694
				B13 Children relationship satisfaction	0.0145	0.0711
Social participation	leisure and entertainment	0.0447	0.3035	C1 Interacting with friends	0.0138	0.1110
				C2 Board games	0.0134	0.1082
	Developmental activities	0.0389	0.2637	C3 Club activities	0.0091	0.0737
				C4 Voluntary activities	0.0094	0.0757
				C5 Attending educational courses	0.0125	0.1010
				C6 Stock investment	0.0136	0.1096
				C7 Using the Internet	0.0150	0.1210
	Caregiving behavior	0.0221	0.1502	C8 Caring for patients	0.0051	0.0413
				C9 Caring for the injured	0.0072	0.0581
	Consumption	0.0417	0.2827	C10 Expenditure for food	0.0128	0.1031
				C11 Other non-food expenses	0.0120	0.0972
Economic status	Wage income	0.0292	0.1978	D1 Salary	0.0101	0.0850
				D2 Fringe benefit	0.0085	0.0714
	Expendable fixed assets	0.0373	0.2530	D3 House property	0.0120	0.1007
				D4 Vehicle	0.0086	0.0723
				D5 Non-financial assets	0.0148	0.1243
	Economic fixed assets	0.0370	0.2512	D6 Farming tools	0.0106	0.0894
				D7 Rental income	0.0109	0.0923
	Financial wealth	0.0439	0.2980	D8 Cash deposit	0.0121	0.1016
				D9 Equity fund	0.0154	0.1302
				D10 Treasury bond	0.0158	0.1328
Physical environment	Infrastructure	0.0513	0.3210	E1 Water resource	0.0157	0.1149
				E2 Bathing facilities	0.0140	0.1024
				E3 Toilets	0.0147	0.1072
	Living facilities	0.0557	0.3485	E4 Elevators	0.0140	0.1022
				E5 Heating facilities	0.0159	0.1159
				E6 Gas or natural gas	0.0159	0.1164
				E7 Air cleaner	0.0145	0.1058
	Living environment	0.0528	0.3304	E8 Tidiness	0.0153	0.1118
				E9 Temperature	0.0169	0.1233
Social security	Basic safeguard	0.0506	0.3636	F1 Pension for Public Servants, Public Institution Employees, and Basic Pension for Enterprise Employees	0.0138	0.1061
				F2 Urban and Rural Resident Pension, New Rural Resident Pension and Urban Resident Pension	0.0142	0.1094
				F3 Urban employee medical insurance	0.0136	0.1046
				F4 Urban and rural resident medical insurance, Urban resident medical insurance, and New rural cooperative medical insurance	0.0158	0.1217
	Additional coverage	0.0392	0.2815	F5 Commercial pension insurance	0.0128	0.0987
				F6 Life insurance	0.0124	0.0957
				F7 Commercial medical insurance	0.0086	0.0667
	Medical service	0.0494	0.3548	F8 Hospitalization	0.0115	0.0885
				F9 Outpatient care	0.0114	0.0882
				F10 Physical examination	0.0156	0.1204

### 3.2 Research methods

#### 3.2.1 Entropy method

The entropy method, as a typical objective assignment technique, can effectively avoid the influence of individual subjective biases. This study utilizes AAI-CHN to calculate the comprehensive and dimension weights for each subsystem and elemental index. By combining these weights with index values, we derived the active aging levels for each province, as well as the levels across six dimensions of active aging, for the years 2011–2018. The scores range from 0 to 100 and are based on a passing benchmark of 60, where higher scores correspond to higher levels of active aging and vice versa. On one hand, these scores reflect the partial current state of active aging development in China at a macro level. On the other hand, it can provide essential data support for further research. The specific process follows the methodology outlined by Luo and Zhou (45).

#### 3.2.2 Dagum Gini coefficient

The Dagum Gini coefficient is a specialized method for depicting and interpreting the magnitude of differences. It not only measures overall regional disparities but also reveals the effects of intra-regional, inter-regional, and transvariation densities on these disparities. This study employs the Dagum Gini coefficient to assess the magnitude and sources of regional differences in active aging within China. The aim is to understand the basic information of the development of active aging in China at the level of relative differences. According to international standards, cut-off points of 0.2, 0.3, 0.4, and 0.5 indicate very low, low, moderate, high, and very high differences, respectively. For the calculation process, please refer to Chen and Jin (46).

#### 3.2.3 Moran index

The Moran Index is a specialized method for measuring the degree of spatial data correlation. It offers advantages such as quantifying autocorrelation, testing the significance of the correlation, and conducting both global and local analyses. This study uses the global Moran Index to test for spatial correlation in the development level of active aging across China. Additionally, the local Moran Index is employed to identify the spatial aggregation patterns of each province. These characteristics will form the basis for this paper’s analysis of active aging from a spatial perspective. For the calculation procedure, refer to Zhou and Wen (47).

#### 3.2.4 Kernel density estimation

The kernel density estimation method is a nonparametric technique used to reveal the distribution trend of a target object over time. It is primarily employed in spatial disequilibrium analysis. To obtain dynamic information on the absolute differences in the level of active aging across the country and its regions, the study employs this method. It examines the distribution location, dynamics, and ductility of active aging levels during the sample period and illustrates the trend of the dynamic evolution of its spatial distribution. For the specific measurement process, refer to Mao et al. (48).

### 3.3 Data source

The data for this study come from the China Health and Retirement Longitudinal Study (CHARLS). CHARLS is a household survey conducted by the National Development Research Institute of Peking University (NDRI), using multi-stage PPS (Probability Proportional to Size) sampling. It covers 28 provincial-level administrative regions in China, excluding Tibet, Hainan, Ningxia, Hong Kong, Macao, and Taiwan. The survey includes 150 county-level units and 450 village-level units, focusing on middle-aged and older adult individuals aged 45 and above. The CHARLS questionnaire design was modeled after the American Aging Population Health and Retirement Study. The CHARLS survey is highly regarded for its response rate and data quality, achieving some of the highest standards globally for similar research. It has received widespread academic recognition (49) and approval from the Biomedical Ethics Committee of Peking University.^1^ CHARLS data collection began with the baseline survey in 2011, and follow-up surveys were conducted in 2013, 2015, 2018, and 2020. Due to the COVID-19-specific survey conducted in 2020, those results are not yet considered in this study. In data processing, samples with missing key information (e.g., gender, age, education) and those aged below 60 years were excluded. When original data had varying degrees of missing values, the mean method was used to impute the missing information.

## 4 Results and analysis

### 4.1 Measurement of the level of active aging

**Figure 3:**
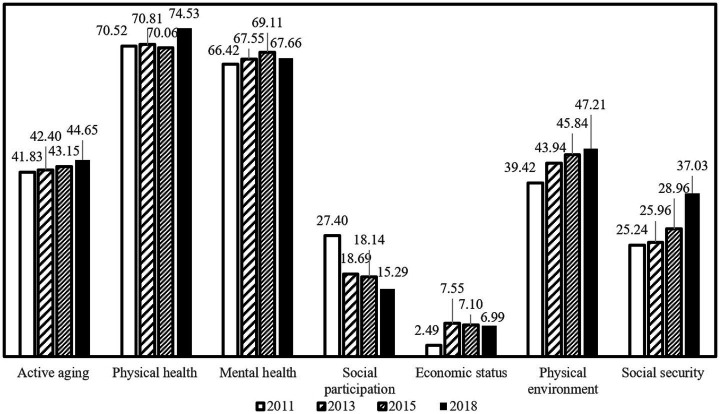
Overall and dimensional development levels of active aging in China from 2011 to 2018.

**Figure 4:**
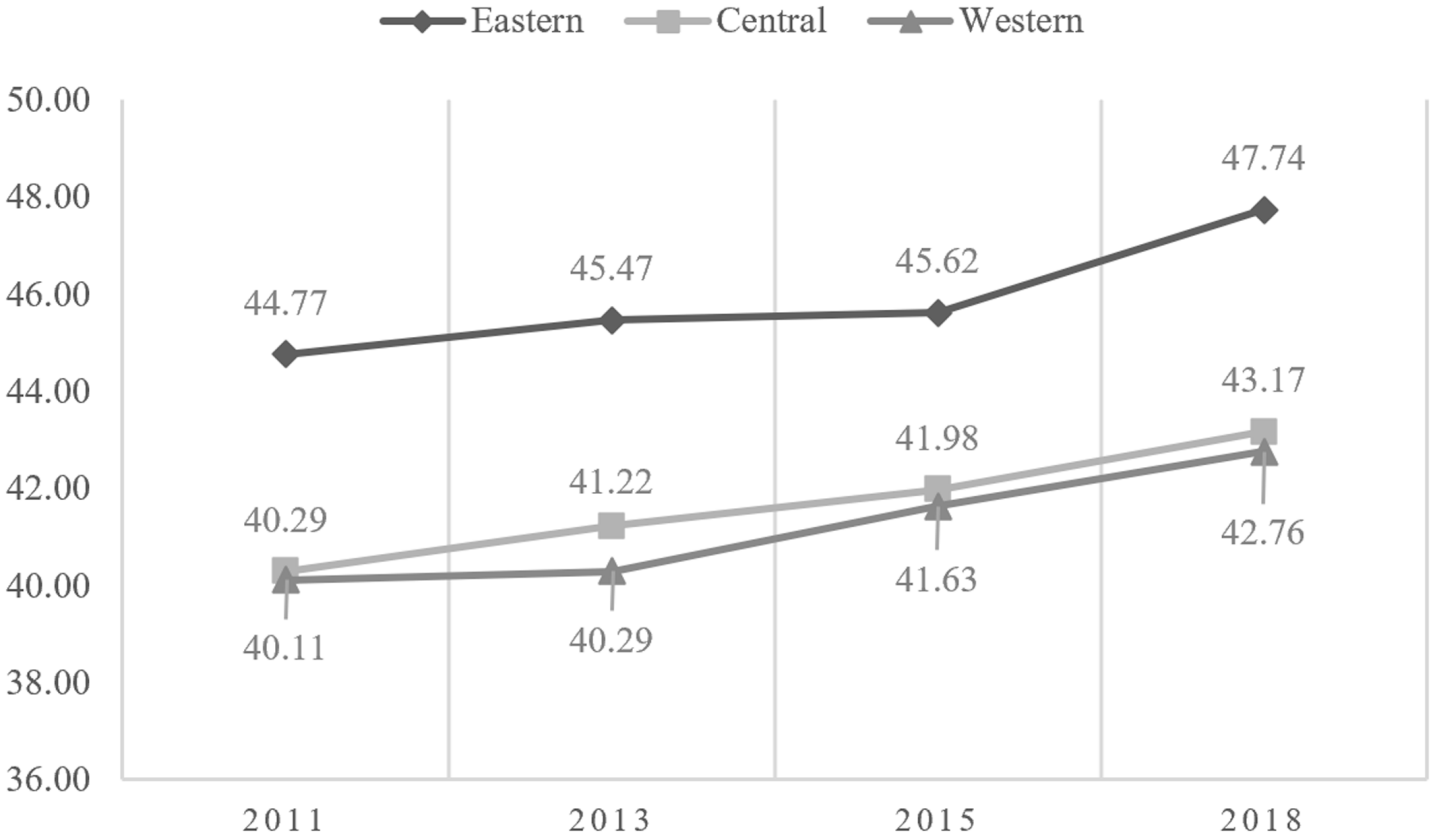
Trends in the level of active aging in the three regions.

**Figure 5:**
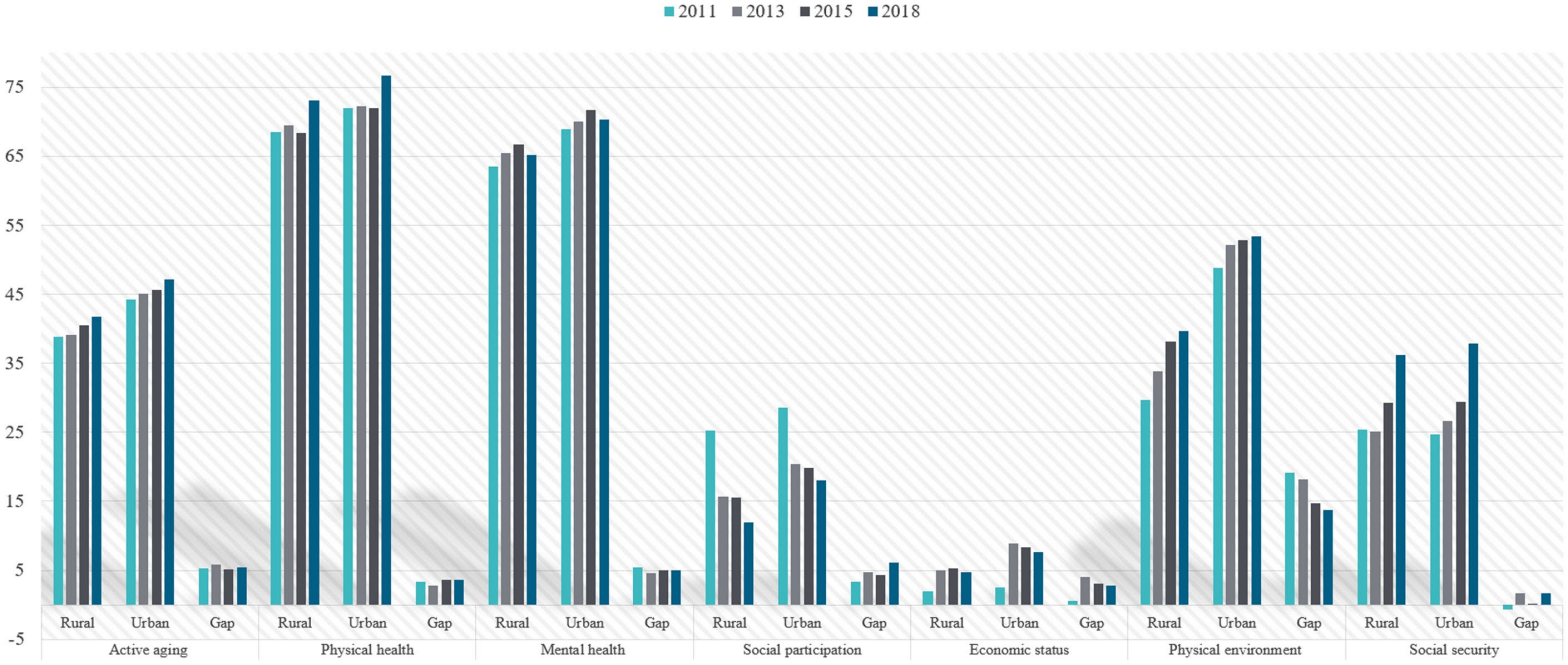
Active aging and its dimension urban–rural development level and urban–rural gap in China from 2011 to 2018.

Based on the indicator weights and values, we provide a basic description of the level of active aging in China over the past ten years. The results are presented in Figures 3–5 and Table 2.

**Table 2:** Typological distribution of the three major regions of active aging in China.

Type	Eastern	Central	Western
Leading provinces	Shanghai, Beijing, Tianjin, Zhejiang, Jiangsu		
Progress provinces	Guangdong	Heilongjiang	Shaanxi, Guangxi
Catching up provinces	Shandong, Fujian, Liaoning, Hebei	Jiangxi, Henan, Hunan, Hubei, Jilin	Xinjiang, Neimenggu, Guizhou
Lagging provinces		Shanxi, Anhui	Yunnan, Chongqing, Sichuan, Gansu, Qinghai

#### 4.1.1 Comprehensive level and level of each dimension

Using the entropy method, we calculated the trend of the overall development level of active aging in China from 2011 to 2018. The results are shown in Figure 3. The mean value of the overall development level of active aging in China ranges from 41.83 to 44.65 (on a scale of 0–100), indicating a lower level of development. However, there is an increasing trend over time, with a growth rate of 6.75%.

Figure 3 also presents the development levels of the six dimensions of active aging in China: (1) The physical health of Chinese older adults is the highest among the six dimensions, with values of 70.52, 70.81, 70.06, and 74.53 from 2011 to 2018, respectively. (2) The average mental health score ranges from 66 to 70, with a composite mean value of 67.69, indicating relatively satisfactory mental health among older adults. (3) The physical environment score increased from 39.42 in 2011 to 47.21, ranking third with a national average value of 44.10. This shows increasing importance placed on the living environment of the older adult. (4) Social security improved from 25.24 in 2011 to 37.03 in 2018. Although the overall development level was not high, the situation was favorable, with an average annual growth rate of 6.68%, thanks to the continuous enrichment of social security content and expansion of coverage in China. It shows a trend of continuous growth. (5) The overall mean value of social participation is 19.89. According to Maslow’s Hierarchy of Needs Theory, social participation belongs to the middle and high-level needs. If the lower-level needs are not fulfilled, the motivation and level of social participation will not be effectively activated. This suggests that social participation is an important factor restricting the development of active aging. (6) The mean value of economic status is only 6.03. Although the development is not as good as expected, it increased from 2.49 in 2011 to 6.99 in 2018, with an average annual growth rate of 25.90%. This indicates that, despite the highlighted problem of poverty among the older adult, economic adjustments made by the State, such as the transformation of the economic development mode, financial reforms, comprehensive deepening of reform and opening up, poverty reduction programs, and improvement of the pension system, have directly raised the economic level of the older adult.

#### 4.1.2 Regional level

The study analyzes the changes in active aging levels in the eastern, central, and western regions of China, as depicted in Figure 4. The overall trend indicates a steady increase in active aging across all three regions, suggesting positive growth momentum. Specifically, the average value of active aging in the eastern region is significantly higher than that in the central and western regions, with mean values ranging from 44.77 to 47.74. In contrast, the levels of active aging in the central and western regions are similar, although the central region shows slightly better outcomes than the western region. Therefore, in terms of absolute differences, which is the differences between the mean values, the level of active aging in the eastern region is markedly higher than that in the central and western regions.

#### 4.1.3 Development level of each province

To explore the development of active aging in 28 provinces, this study utilized the findings of He et al. and Wei and Li (50, 51). The national level was categorized into four types based on the mean level (41.83–44.65), the composite mean (E) (43.01), and the standard deviation (SD) (3.65) for each year. Provinces with a composite mean of 44.84 or higher are referred to as “leading provinces.” Those with a composite mean between 43.01 and 44.84 are termed “progressive provinces.” Provinces with a composite mean between 41.19 and 43.01 are labeled “catching up provinces,” and those with a composite mean of less than 41.19 are called “lagging behind provinces.” The distribution of these categories across different regions is illustrated in Table 2.

The leading provinces in coping with aging include Shanghai, Beijing, Tianjin, Zhejiang, and Jiangsu. These regions have achieved remarkable results in actively addressing aging. The progressing provinces consist of Heilongjiang, Guangdong, Shaanxi, and Guangxi, reflecting a high level of active aging. The 12 catching-up provinces are Jiangxi, Xinjiang, Shandong, Fujian, Liaoning, Henan, Hebei, Hunan, Hubei, Inner Mongolia, Guizhou, and Jilin. The level of active aging in these provinces is below the national average, but there is potential for improvement. The remaining seven provinces are lagging behind, indicating a significant gap compared to the leading provinces.

In this evaluation, the leading provinces are primarily concentrated in the eastern region. Among the progressing provinces, one is in the east, one is in the center, and two of them are in the west. For the catching-up provinces, four are in the eastern region, five are in the central region, and three are in the western region. The lagging provinces are mainly in the central and western regions, with two in the central region and five in the western region. This distribution highlights regional differences in China’s active aging development. The eastern region, with its economic advantages, has made notable progress in terms of active aging. In contrast, the central and western regions, though currently behind, are showing positive momentum. Some areas have achieved higher success in active aging due to their accumulated resources and potential.

#### 4.1.4 Urban and rural development levels of active aging

The study analyzed the development levels, trends, and urban–rural differences of active aging from 2011 to 2018. Overall, the level of active aging among the urban older adult was 45.54 and that of rural older adult was 40.06 from 2011 to 2018. Although the level of active aging among urban older adult was higher than that of rural older adult, the average difference was only 5 points, indicating that there was no significant fluctuation in the urban–rural difference during the eight years.

Specifically, (1) From 2011 to 2018, the physical health level of the older adult in both urban and rural areas improved, with a small and stable urban–rural gap. (2) The mental health level of the older adult in urban and rural areas showed a small gap, and the urban–rural difference in 2018 was slightly narrowed compared to the baseline year. (3) Compared to rural areas, the level of social participation among urban older adult was slightly higher. In 2011, the level of social participation among the older adult in both urban and rural areas was much higher than in other years, and the urban–rural gap widened in 2018 compared to 2011. (4) In 2011, the economic levels of the older adult in both urban and rural areas were low and almost identical, but the improvement in the economic level of urban older adult in 2013 led to an expansion of the urban–rural gap, which subsequently narrowed year by year. (5) Among the six dimensions, the physical environment showed the largest difference between urban and rural older adult, with an average gap of 16.45, but this gap continued to narrow over time. (6) In 2011, the level of social security for rural older adult was slightly higher than that of urban older adult, but the level of urban older adult surpassed rural older adult in 2013. In 2015, development showed a balanced trend between urban and rural areas, and in 2018, the urban level was again higher than that of the rural area (as shown in Figure 5).

### 4.2 Regional differences in active aging

The study identified absolute differences in active aging across the eastern, central, and western regions of China. Subsequently, the research will utilize the Dagum Gini Coefficient to analyze the evolutionary characteristics of these relative differences in active aging.

#### 4.2.1 Overall differences and sources

**Figure 6:**
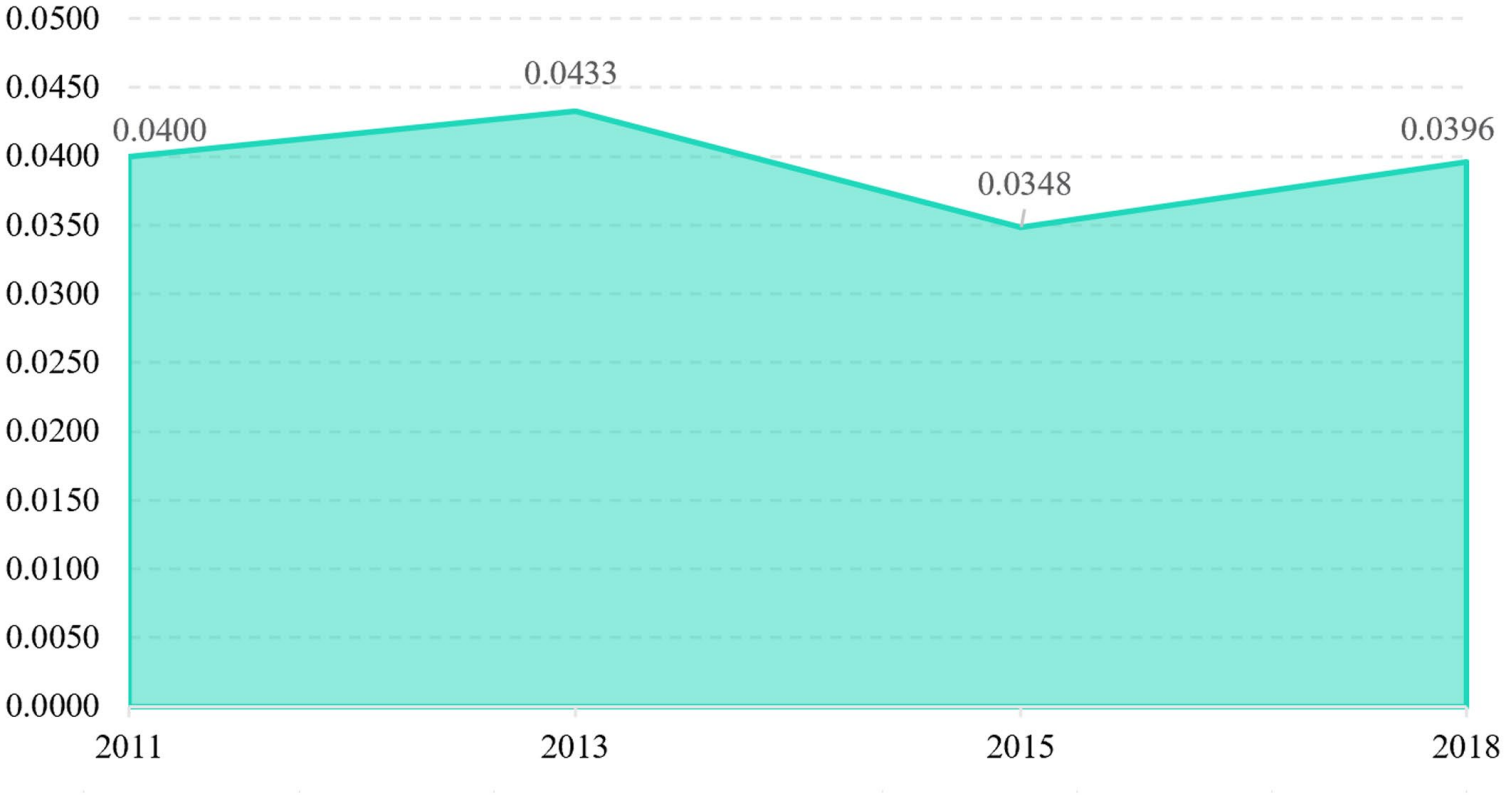
Overall differences in active aging levels in China from 2011 to 2018.

Figure 6 displays the result of overall Gini coefficient for active aging in China from 2011 to 2018. In general, the average Gini coefficient for the degree of active aging is 0.0394, falling below the international standard threshold of 0.2. This suggests that the overall disparity in active aging throughout the country is relatively minimal. Examining specific years, the Gini coefficient rose from 0.0400 to 0.0433 between 2011 and 2013, decreased to 0.0348 in 2015, and increased slightly to 0.0396 in 2018. This suggests that the overall variation in active aging development remained largely consistent.

**Figure 7:**
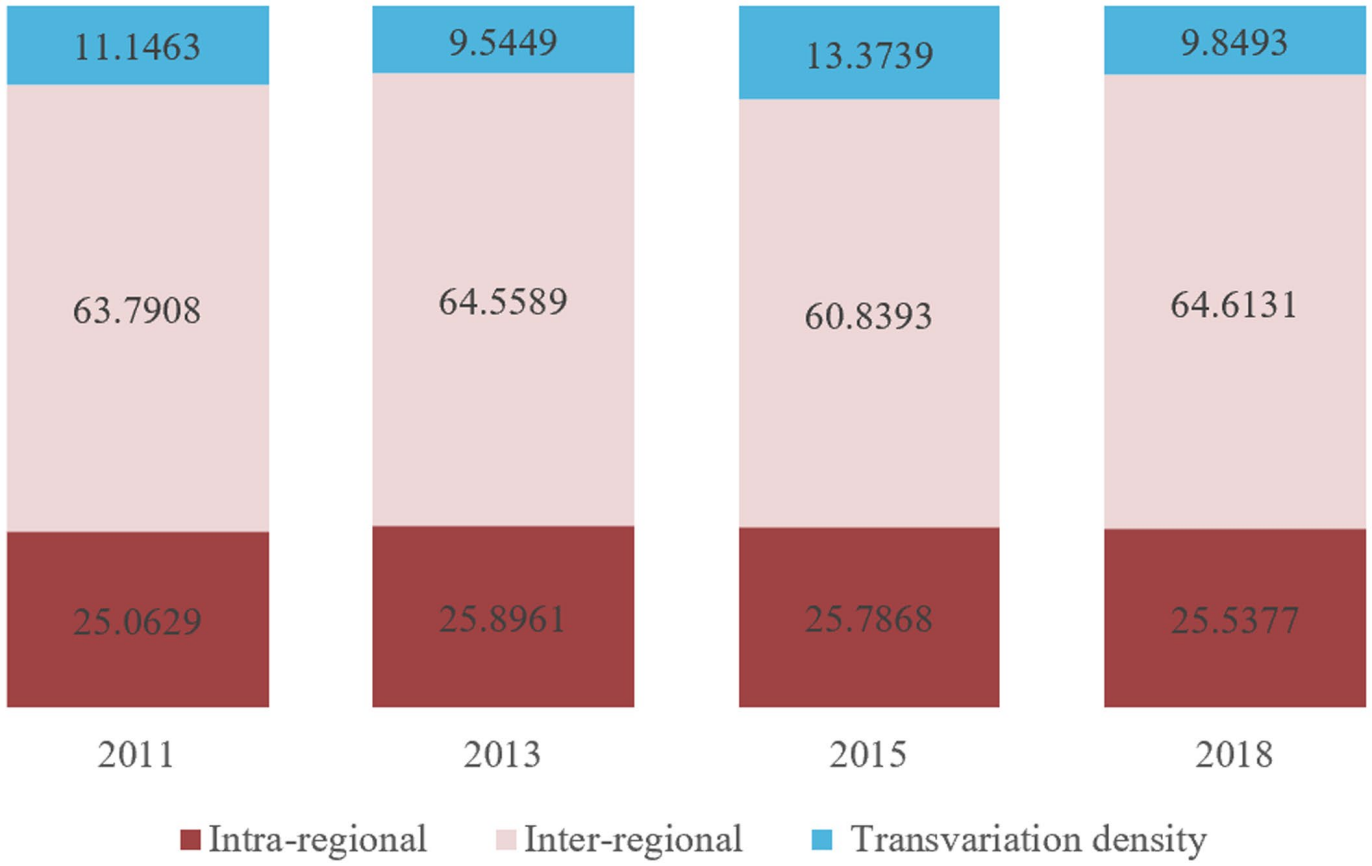
Contribution to regional differences in active aging levels in China from 2011 to 2018.

Regarding the structure of the overall difference, the contribution rates of inter-regional and transvariation density exhibited alternating fluctuation patterns over the sample period, characterized as “rising-declining” and “falling-rising,” respectively. The average contribution rates are as follows: intra-regional variation at 25.57%, transvariation density at 10.98%, and inter-regional variation, which is the highest, at 63.45%. This indicates that the overall disparity in active aging in China is primarily driven by inter-regional differences, followed by intra-regional differences, with transvariation density contributing the least (see Figure 7).

#### 4.2.2 Intra-regional differences

**Figure 8:**
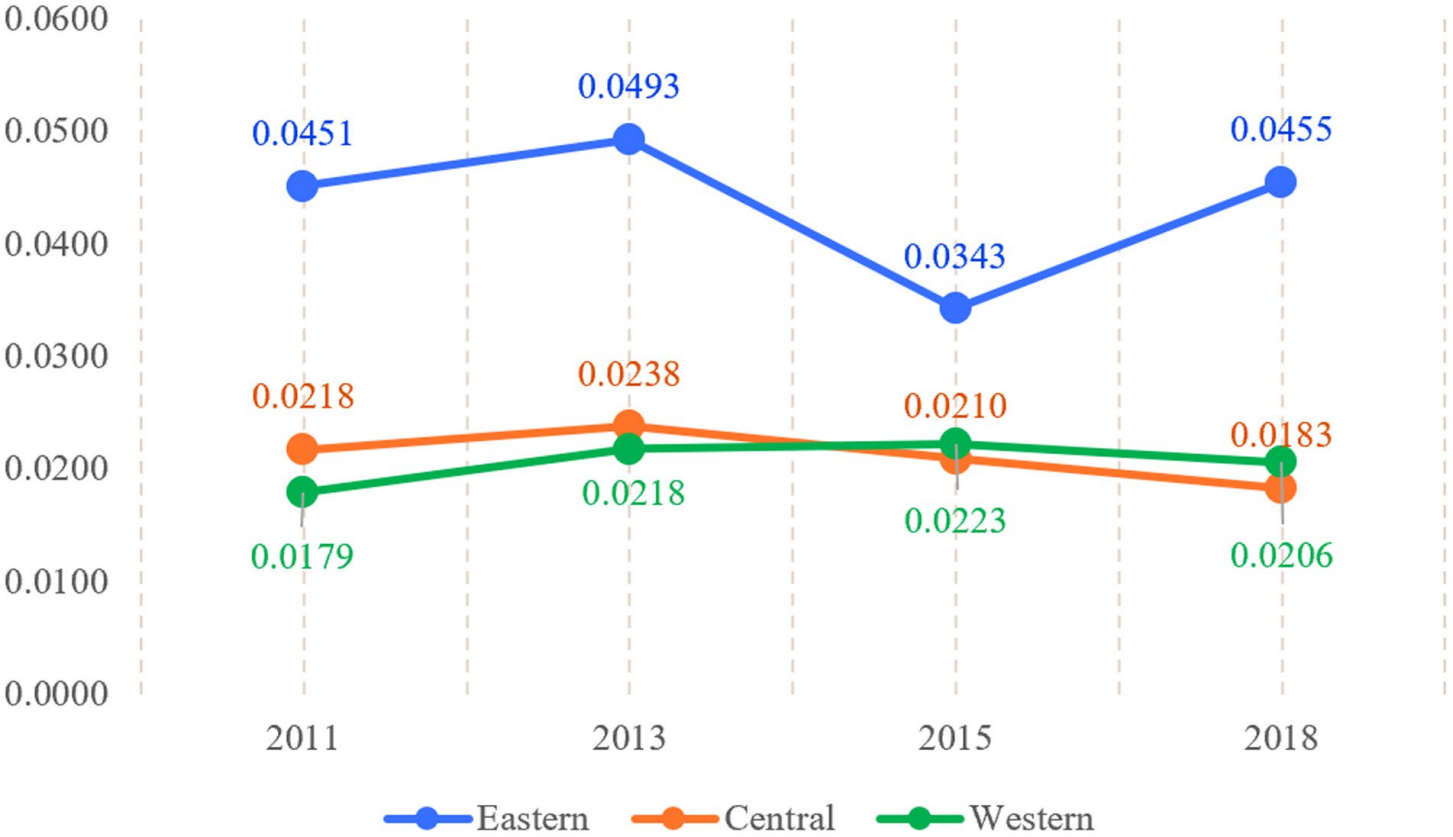
Intra-regional differences in active aging levels in China from 2011 to 2018.

Figure 8 illustrates the Gini coefficient results for active aging levels in the East, Central, and West regions from 2011 to 2018. The eastern region exhibits the highest mean Gini coefficient at 0.0435, indicating unbalanced development in active aging levels within this region. This disparity is due to significant differences in active aging levels among provinces like Beijing, Shanghai, Tianjin, Hebei, Jiangsu, Zhejiang, Fujian, Shandong, Liaoning, and Guangdong, with mean levels ranging from 43.40 to 54.90. In contrast, the central region’s mean Gini coefficient is 0.0212, showing a “small increase and then gradual decrease” pattern, which suggests diminishing disparities in active aging levels within the region. Notably, the central region is the only one showing a decreasing trend in intra-regional differences. The western region has an average Gini coefficient of 0.0207, indicating smaller intra-regional differences. Despite slight fluctuations from 2011 to 2018, the overall trend in the western region’s active aging levels has been a slow increase, likely due to improvements in areas such as Shaanxi and Guangxi.

#### 4.2.3 Inter-regional differences

**Figure 9:**
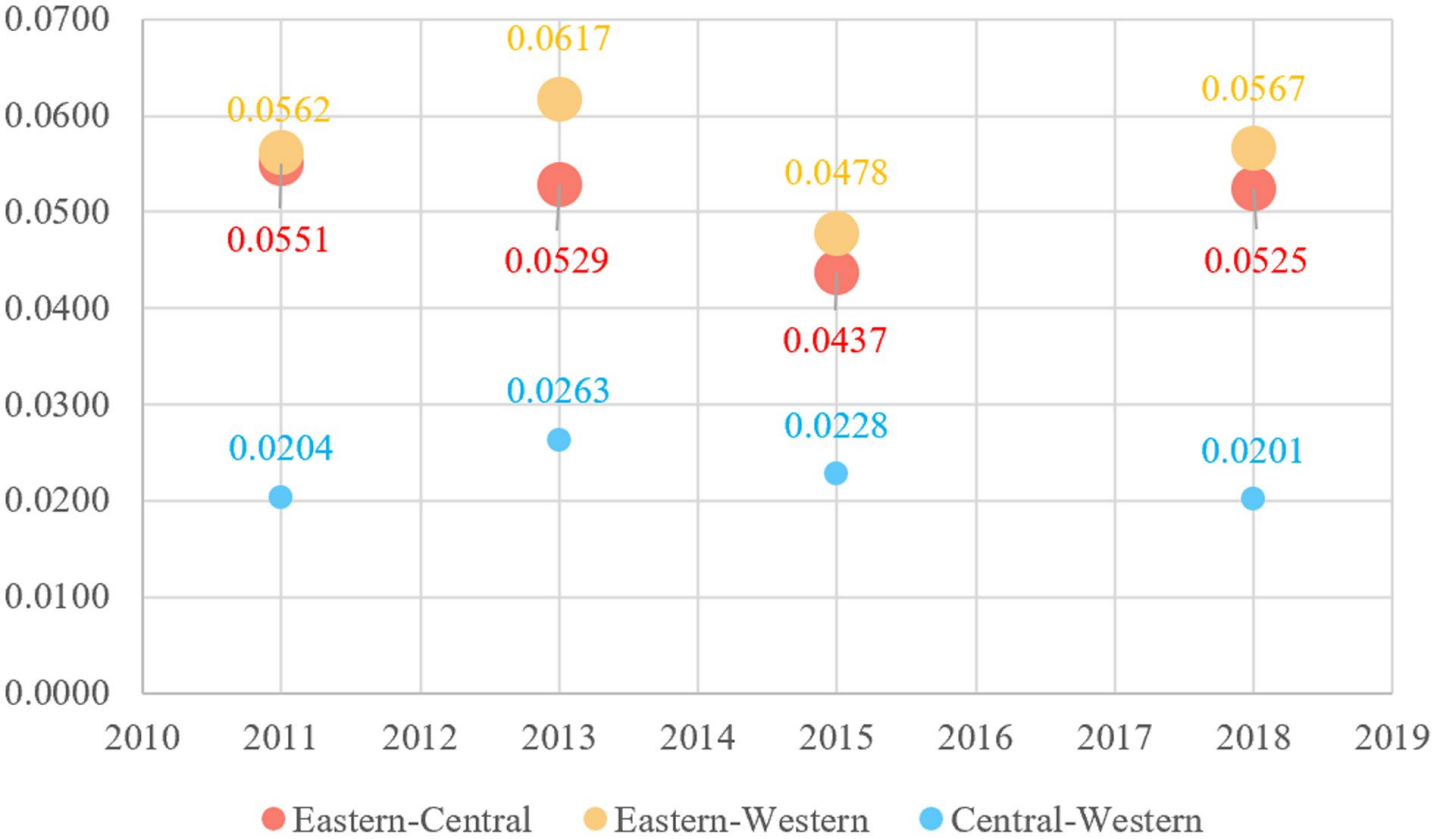
Inter-regional differences in active aging levels in China from 2011 to 2018.

Figure 9 depicts the interregional differences in active aging development levels and their trends from 2011 to 2018. The mean interregional Gini coefficients between the East and West, and between the East and Central regions is higher, which both exceed 0.05. Notably, while the differences between the East and West widened after fluctuations in the sample period, the gaps between the East and Central narrowed over the same period. In contrast, the mean interregional Gini coefficient between the Central and Western regions is the lowest. The mean is 0.0224, indicating smaller differences between these two regions. Overall, the differences between the eastern region and the other regions are more pronounced.

### 4.3 Spatial correlation of active aging

To analyze the spatial distribution and space aggregation of active aging, we utilize the global Moran’s index and local Moran’s index in this section.

#### 4.3.1 Global spatial autocorrelation

Table 3 shows that the Moran Index from 2011 to 2018 is consistently positive, with a significance level below 5%. This signifies a significant spatial correlation in active aging levels among provinces, indicating mutual provincial influence. For instance, Beijing and Tianjin, which are the driving forces, significantly drive the growth of active aging in Hebei Province. The province’s growth rate reached 6.85% between 2011 and 2018, ranking third nationally.

**Table 3:** Global spatial autocorrelation Moran’s index of active aging levels in China.

Year	2011	2013	2015	2018
Moran′s *I*	0.200^**^	0.242^**^	0.172^**^	0.235^**^
Z-value	2.063	2.416	1.783	2.301
Relevance	Positive correlation			
Spatiality	Aggregated distribution			

***, **, * denote significance levels of 1, 5 and 10 percent, respectively.

The trend in the global Moran Index reveals that China’s active aging levels have undergone phases of “rising, then falling, then rising,” reflecting an overall increase in spatial correlation despite fluctuations. This indicates that provinces with higher levels of active aging tend to cluster together. Consequently, this finding highlights the spatial pattern of active aging development and serves as a crucial reference for policy formulation.

#### 4.3.2 Local spatial autocorrelation

**Figure 10:**
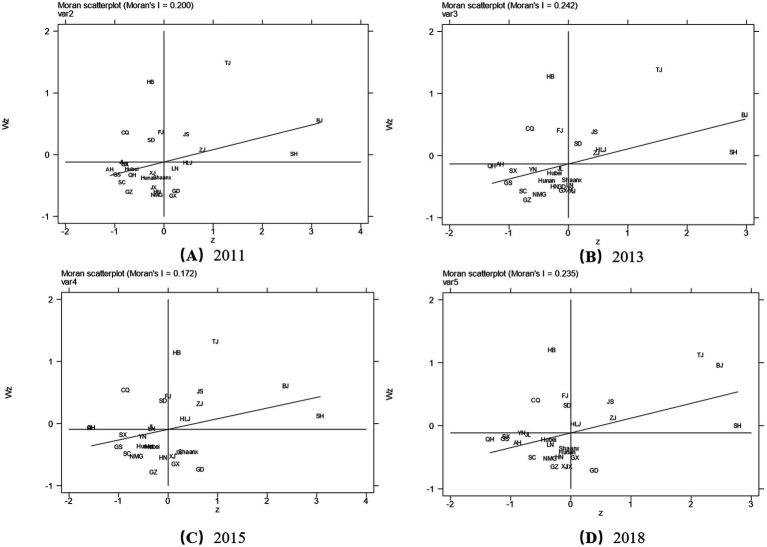
**(A–D)** Local Moran’s index scatter plot of active aging levels by province.

Figures 10A–D illustrate the distribution of active aging levels in Chinese provinces, categorized into four quadrants based on local Moran index analysis from 2011 to 2018. These quadrants represent “High-High,” “Low-Low,” “High-Low,” and “Low-High” aggregation patterns. The majority of provinces are in the third quadrant, indicating a “L-L” pattern. Meanwhile, the Beijing-Tianjin-Hebei and Jiangsu-Zhejiang-Shanghai regions are mostly in the first quadrant, showing an “H-H” pattern.

Specifically, the eastern provinces of Tianjin, Beijing, Shanghai, Jiangsu, and Zhejiang consistently appear in the first quadrant, indicating higher levels of development for themselves and their neighboring provinces. In contrast, the western provinces are predominantly in the third quadrant, indicating lower levels of development for themselves and their neighboring provinces. Hebei, Chongqing, Fujian, and Shandong are mostly in the second quadrant, representing lower self-development levels but higher levels in neighboring provinces. This may be due to more rational and scientific active aging development planning in neighboring areas. Guangdong and Guangxi, however, frequently appear in the fourth quadrant, showing higher development for themselves but lower development in neighboring provinces.

Regarding the migration of spatial correlation patterns, most provinces show stable spatial correlation with minimal changes. However, some eastern and western provinces have transitioned between neighboring quadrants. For example, Shandong and Hebei moved between the first and second quadrants, while Liaoning, Guangxi, and Shaanxi transitioned between the third and fourth quadrants.

### 4.4 Dynamic evolution of active aging

Although the Dagum Gini coefficient captures the size and source of relative differences in active aging levels across the country and its three major regions, it does not reflect the absolute differences and their dynamics. Hence, this section will evaluate the spatial distribution and dynamic evolution of active aging levels in China from 2011 to 2018.

#### 4.4.1 National active aging density estimation

**Figure 11:**
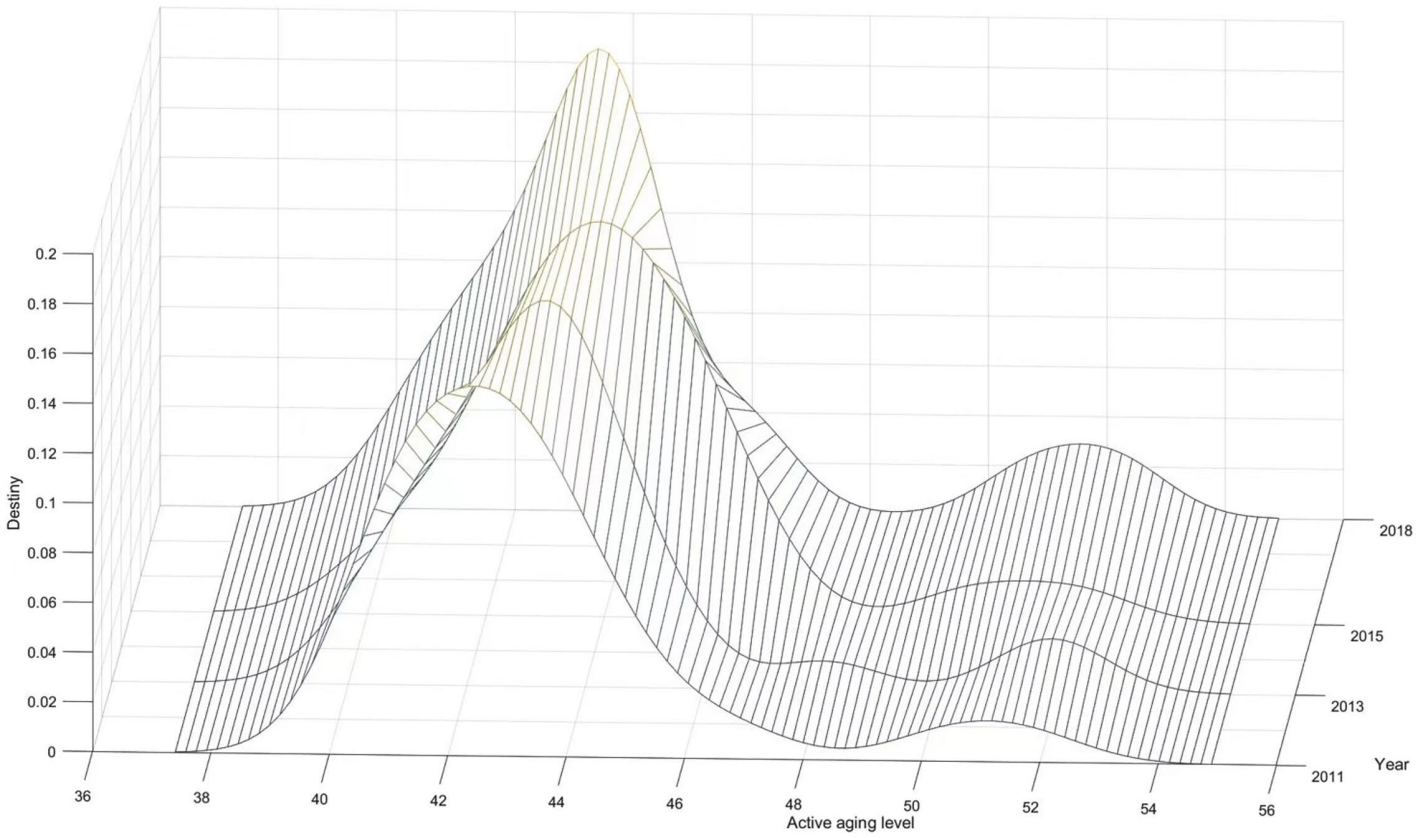
Dynamic evolution of active aging levels in China.

Using the Kernel density estimation method, we selected data on the active aging levels of 28 provinces in China for the years 2011, 2013, 2015, and 2018, and plotted the dynamic evolution trend (see Figure 11). The position of the curve shows that the center of the Kernel density curve has shifted to the right obviously, indicating an annual increase in the level of active aging in China. The width of the main peak in 2018 contracted compared to that in 2011, reflecting increased aggregation in the active aging levels of the 28 provinces. Regarding distribution ductility, the national distribution curve shows an obvious right tail, indicating provinces with higher levels of active aging, such as Beijing, Shanghai, and Tianjin. Additionally, the Kernel density curve for active aging levels displayed a double-peak phenomenon between 2011 and 2018. This double-peak became increasingly evident over time, reflecting a gradual intensification of the polarization of active aging across the country.

#### 4.4.2 Density estimation of active aging in three major regions

**Figure 12:**
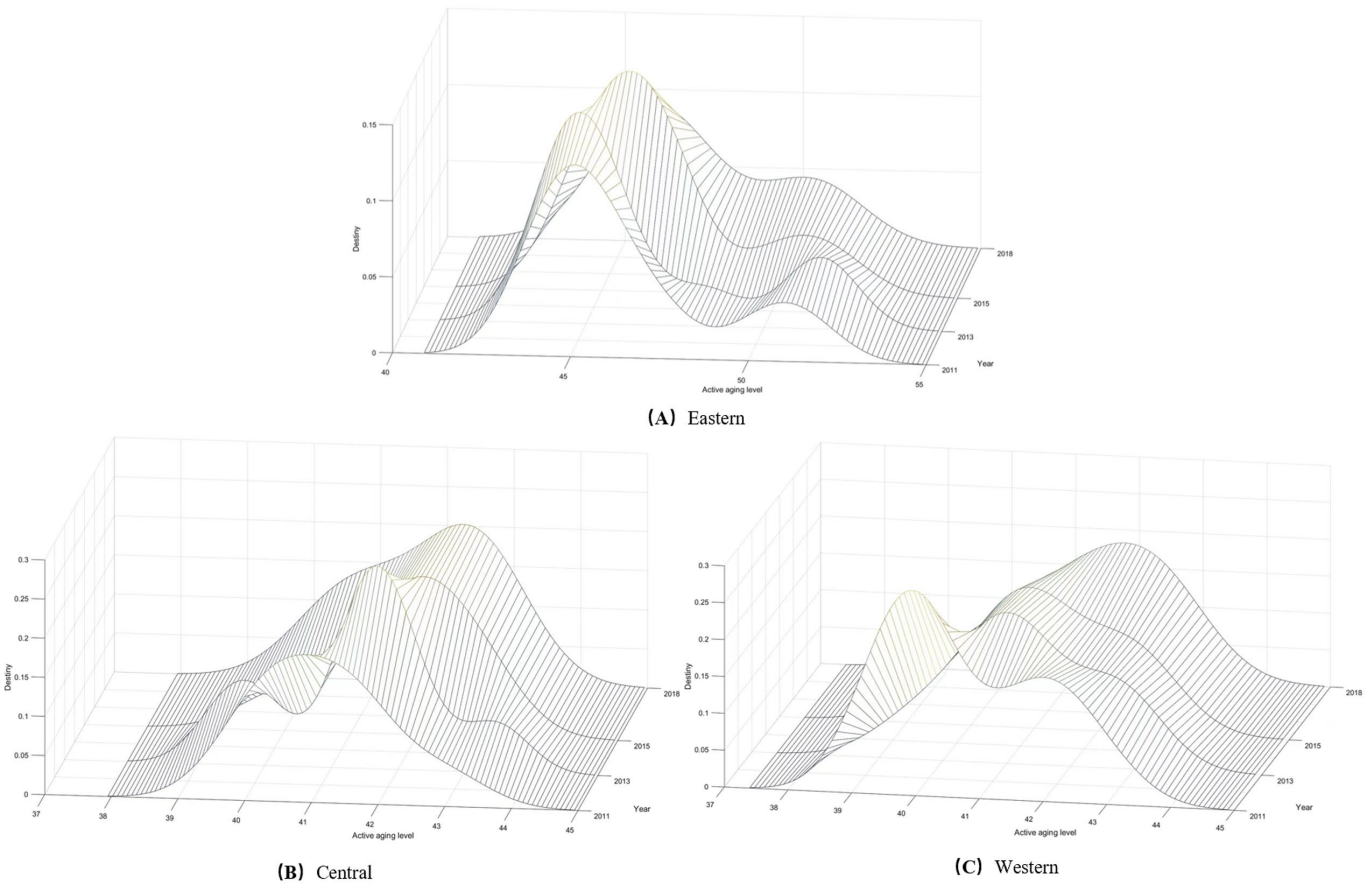
**(A–C)** Dynamic evolution of active aging levels in the three major regions of China.

Figures 12A–C respectively illustrate the Kernel density maps of the eastern, central, and western regions of China. Through observation, the position of the main peak in the active aging distribution curve for all three regions shifted to the right from 2011 to 2018. This shift indicates an increased level of active aging across these regions.

Regarding distributional ductility, the eastern region exhibits a right-tail phenomenon, whereas the central and western regions do not. This observation suggests uneven development in the eastern region, primarily due to higher levels of active aging in provinces like Beijing, Shanghai, and Tianjin. In contrast, active aging development in the central and western regions appears more balanced.

In terms of distributional polarization, the eastern region’s Kernel density curve exhibited a double-peak pattern from 2011 to 2018. This pattern indicates a two-tiered polarization of active aging levels of the eastern region, particularly in 2013, when a more prominent sub-peak emerged. The central region displayed a multi-peak phenomenon in the Kernel density curve in 2013, but this two-tier differentiation weakened after 2013. In the western region, the Kernel density formed two main peaks in 2011, suggesting that there was a differentiation; however, this phenomenon has improved over time.

## 5 Robustness test

To avoid potential biases in empirical results caused by the choice of measurement methods, this paper conducts robustness checks by altering the measurement of the China Active Aging Index.

**Figure 13:**
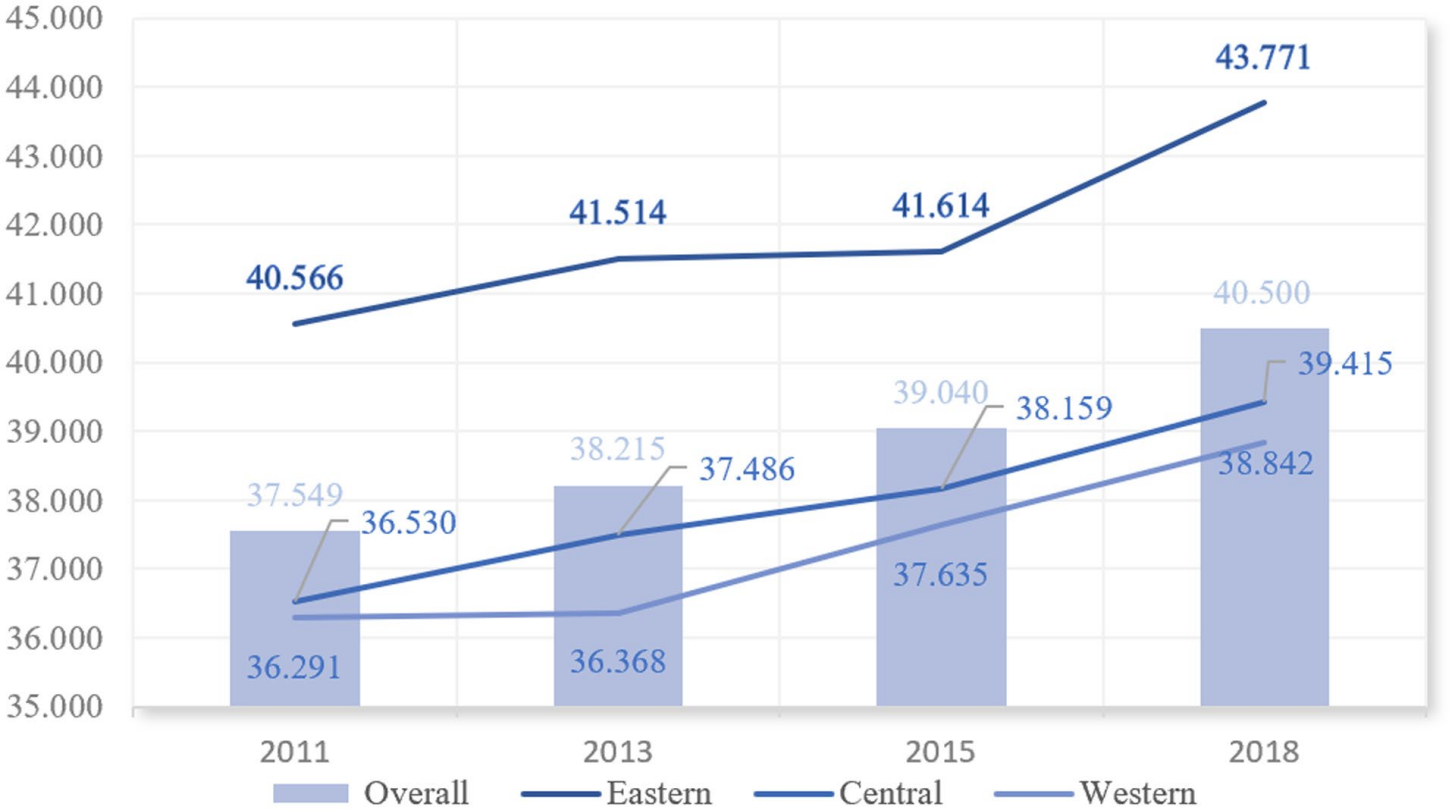
Overall and regional trends of active aging in China.

**Figure 14:**
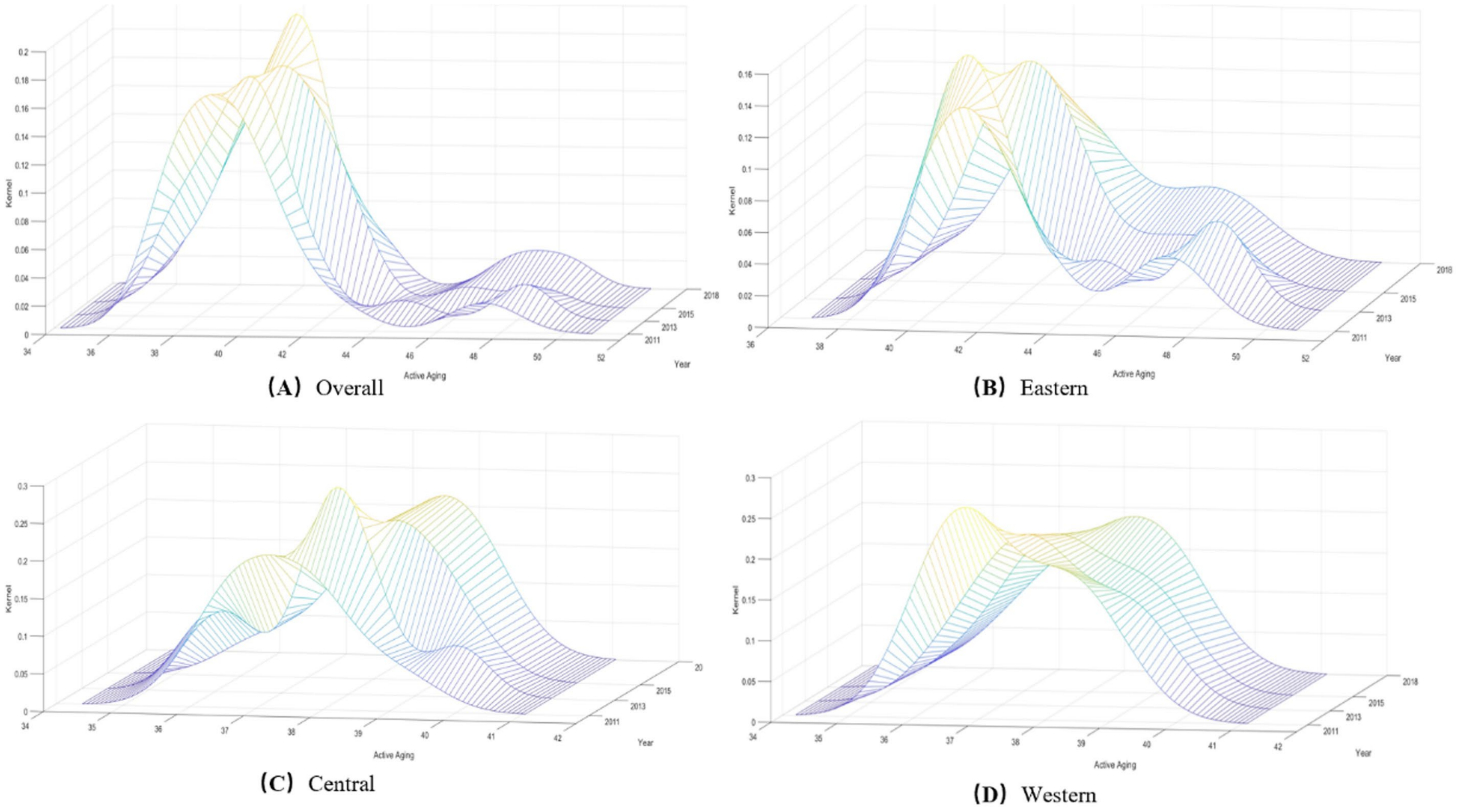
**(A–D)** Dynamic evolution of active aging across China and its regions.

**Figure 15:**
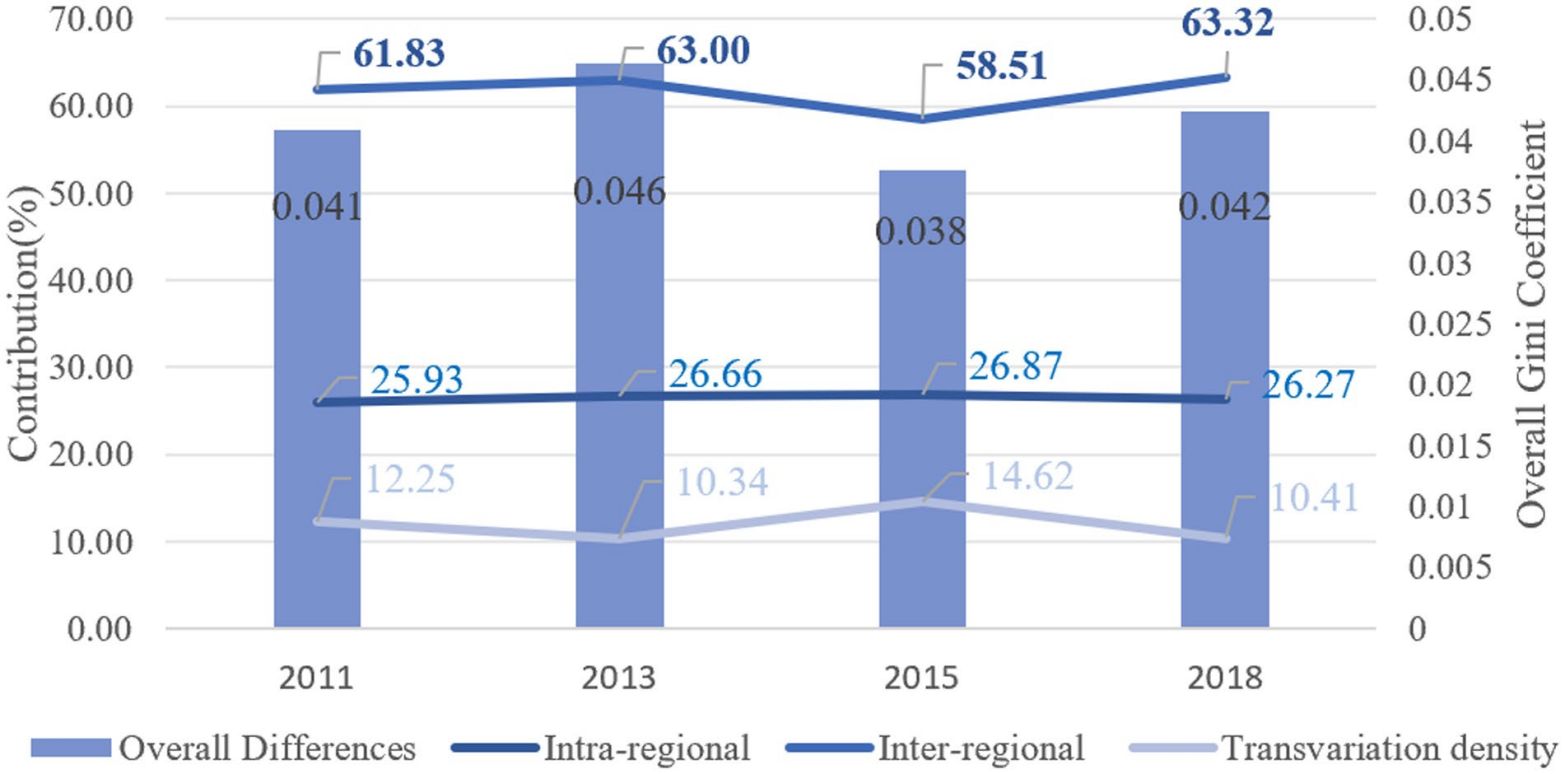
Overall Gini coefficient and decomposition results of regional difference contribution rate for active aging in China.

Firstly, we change the method for determining indicator weights. In this study, we mainly obtain the weights of the indicators using the entropy method, which is an objective weighting approach. However, for the robustness test, we adopt the equal weighting method, a widely used subjective weighting approach, to determine the weights and subsequently calculate the level of active aging in China from 2011 to 2018. Then, we use this result to conduct secondary verification of the research findings through methods such as Dagum Gini Coefficient, Moran’s Index, and Kernel Density Estimation. As shown in Figures 13–15, although there are some numerical changes in the recalculated level of active aging in China, the overall trends, dynamic evolution, and regional differences at both the national and regional levels remain obviously unchanged. Additionally, the global Moran’s I values for the period from 2011 to 2018 are 0.183 (*p* = 0.027), 0.235 (*p* = 0.009), 0.159 (*p* = 0.045), and 0.228 (*p* = 0.012), and there are no fundamental changes in their significance or trends. Therefore, the research findings presented above are relatively robust.

Secondly, the synthesis method for the composite index was changed. In this paper, we primarily use the linear weighting method to synthesize the China Active Aging Index. For robustness test, we adopted the Technique for Order Preference by Similarity to Ideal Solution (TOPSIS) to synthesize the index and then calculated the China Active Aging Index for the period 2011–2018. Due to limited space, specific research data is not presented here. The results show that they are generally consistent with previous findings, indicating that the research conclusions have a certain degree of robustness.

## 6 Conclusion and discussion

This study, based on a comprehensive understanding of active aging in China, constructed an AAI-CHN encompassing six key dimensions: physical health, mental health, social participation, economic status, physical environment, and social security. Utilizing CHARLS data, this study quantitatively describes the current situation, regional differences, spatial correlations, and spatial–temporal evolution of active aging in China. These insights aim to provide essential facts and guidance for the development and promotion of active aging in China. The study concludes the following points:

(1) On the whole, the level of active aging in China is still relatively low, but looking at the data from 2011 to 2018, it shows a stable year-by-year increasing trend. In the multidimensional assessment of active aging, we find that the physical and mental health of the older adult constitute the core driving force for promoting active aging, followed by improvements of material environment and social security. However, social participation and economic status have become the significant shortcomings in enhancing the level of active aging.

Further analysis is as follows: Firstly, in recent years, the Chinese government has issued several policies related to older adult care, which are about medical care, older adult care, cultural activities, education, and community organizations. These policies aim to benefit more older adult people from the reform and development. As the policies are continuously implemented, their effects are becoming more apparent year by year, and their role in promoting active aging among the older adult is increasingly strengthening. Secondly, many studies indicate that areas that the older adult can control themselves, such as physical and mental health, and family environment, perform relatively well. On the contrary, the levels of dimensions that are more influenced by cultural factors, social customs, and traditional values, such as social participation and economic status, are relatively low. For example, there is still thought in Chinese society that “the old are useless,” which leads to the older adult often being forced to withdraw from economic and social activities after getting old. This phenomenon urgently needs to be changed. Therefore, the development of active aging not only requires the guiding role of formal institutions but also the influence of informal institutions.

(2) When considering regional differences, inter-regional disparities are the primary source of variation in the level of active aging in China. Notably, the disparities between the eastern region and other regions are particularly significant, whereas the differences between the central and western regions are relatively minor.

The driving forces behind this phenomenon are complex and diverse. It is evident that economic development is closely related to local finance and wage levels, which directly determine the investment in older adult care facilities, the allocation of medical resources, and the income levels of residents. These factors, in turn, enhance the level of active aging among the older adult in the eastern region and gradually widen the gap with other areas. Additionally, the degree of aging in a region and the government’s emphasis on this issue are also crucial factors. For instance, Shanghai established employee pension insurance accounts in 1991 and achieved a high enrollment rate in universities for the older adult in 2008. In 2014, Beijing launched the “Beijing Tong - Elderly and Disabled Assistance Card” to improve the quality of life for the older adult. Furthermore, the relatively lower environmental and living quality in the central and western regions is also a potential factor contributing to the gap in active aging levels between regions. In summary, to fully understand the regional disparities in China’s active aging, it is necessary to consider a multitude of factors, including economic development, policy support, environmental quality, and living quality.

(3) From the perspective of spatial correlation, the level of active aging in China exhibits spatial clustering. Each province can receive and transmit effects, with a spatial clustering trend showing phases of “enhancement, weakening, and enhancement” during the sample period. Moreover, the development of active aging demonstrates localized clustering effects. Notably, “Low-Low” clustering is more frequent and primarily occurs in the central and western regions. In contrast, “High-High” clustering is mainly found in the eastern regions, such as Beijing, Tianjin, Hebei, Jiangsu, Zhejiang, and Shanghai. This indicates the presence of a “neighborhood effect” in the development of active aging, highlighting the importance of spatial factors and coordinated development among neighboring provinces.

Further exploration shows that this spatial agglomeration is not just due to geographical proximity but may also involve several factors: first, it’s related to the regional spread of economic development models, where successful models often spread first to nearby areas; second, it’s closely linked to regional policies and planning, as governments’ regional development strategies may guide resources to focus on specific areas, affecting the spatial distribution of active aging. So, understanding the spatial agglomeration of active aging needs to consider the interactions among geography, economy, policy, and other factors.

(4) From a dynamic evolution perspective, the polarization of active aging in China has gradually intensified. Among them, the development of active aging in the eastern region is uneven, showing a clear two-tier differentiation between 2011 and 2018. In contrast, the central and western regions experienced more balanced development in most years, although there were some instances of divergence.

During the process of analysis, we also considered other possible explanations: The higher levels of active aging in provinces like Beijing, Shanghai, and Tianjin compared to other eastern provinces may explain some of the polarization in eastern regions. However, the uneven development within eastern provinces themselves is also a factor that cannot be ignored. For example, differences in resource allocation, infrastructure, and social welfare between cities and nearby towns may further increase intra-regional polarization.

Accordingly, this study makes several contributions. First, within the international framework, the Active Aging Scale with Chinese characteristics was constructed by combining China’s policy orientation and academic recommendations, and through a rigorous scale development process. Second, this study measures the current status of active aging in 28 Chinese provinces over the past ten years. It includes overall measurements, dimensions, geographic regions (eastern, central, and western region), province, and urban and rural levels. Third, the study analyzes the relative differences in active aging levels and their sources at the national level and in the three major regions. Additionally, it explores the relationship between active aging levels and spatial and geographic regions, showing the changing trends over the past ten years. For China, understanding the current situation of active aging from national, regional, temporal, spatial, dynamic, and static perspectives can provide crucial data for problem-solving, action intervention, and policy formulation. It is also expected to offer research ideas, measurement tools, and action strategies for other countries, especially developing nations.

However, due to limited conditions and perspectives, this study has several shortcomings. Firstly, while striving to scientifically develop the China Active Aging Scale and conduct normative research on its current status, our work was constrained by our reliance on data sources. For example, the 2020 CHARLS survey focused on the COVID-19 pandemic, resulting in the exclusion of many original items, which forced us to abandon the 2020 data. Additionally, the latest data had not been released at the time of our research, limiting our exploration to the years 2011–2018. Furthermore, CHARLS lacks data from Tibet, Hainan, Ningxia, Hong Kong, Macao, and Taiwan, leading to insufficient understanding of these regions. Secondly, due to the complexity of active aging, the selected indicators are not perfect, potentially excluding some content such as chronic diseases, work, medical expenses, and pensions. Thirdly, this paper only analyzes the level measurement, regional differences, and dynamic evolution of active aging in China, without investigating influencing factors or constraints.

These limitations emphasize the necessity for continued research. In the future, we will continue to focus on research on active aging in China and keep abreast of updates to this study as new data emerges. We plan to consider Tibet, Hainan, Ningxia, Hong Kong, Macao, and Taiwan as typical regions for targeted investigations, either through self-administered questionnaires or qualitative research, to understand the level of active aging among the older adult in these regions. Meanwhile, continuous optimization and improvement of the indicator system are essential. Finally, based on the conclusions of this study, we will further expand the research content, such as conducting studies on driving factors, obstacle analysis, development trend prediction, and convergence research.

## 7 Countermeasures

First, optimize the orientation of policies. At the national level, policies aimed at meeting the basic survival needs of the older adult must balance addressing developmental shortcomings and improving quality. Policies aimed at meeting the medium-to-high needs of the older adult should strive to benefit all older adult individuals. For example, in urban areas or areas that older adult-friendly facility renovations are relatively well-developed, the housing and construction department, information technology departments, and local governments can collaborate to create intelligent spaces for the older adult and improve their living environment. In towns, governments can enhance basic service facilities such as activity centers and canteens for the older adult through third-party services and public-private partnerships. It’s worth mentioning the “Dining Hall for the Elderly,” also known as the “Panda Dining Hall” in Shanghai. This is a company located in the heart of the community, specifically catering to the older adult. It has greatly solved the dining problems for older adult people in the nearby areas. In recently poverty-alleviated rural areas, it is crucial to continue updating infrastructure, including water, electricity, gas, heating, toilets, and networks. Additionally, social participation is a critical yet often overlooked developmental component. Based on national policy guidance, more equal opportunities should be provided to enable the older adult nationwide to participate in social, cultural, and recreational activities, which represents a significant approach taken by countries like the United States in response to the aging society.

Secondly, we should address the developmental shortcomings. On the economic front, it is essential to strengthen the basic pension insurance system to ensure older adults have a stable pension source. Tax support should be provided for the development of inclusive financial products that offer low to medium risks, steady returns, and a balance between savings and insurance functions. An in-depth understanding of older adults’ needs regarding their lives, health, and spirituality is necessary to build a system of multilevel, personalized products and services, thereby stimulating their consumption potential. Lastly, companies should be encouraged to fulfill their social responsibilities by retaining aged workers whenever possible. For instance, some large North American enterprises strive to help aged employees acquire new knowledge and skills, thus maximize the retention of experienced staff. In terms of participation, society should not only dismantle the inherent mindset of “the theory of the burden of the elderly” and encourage older adults to utilize their potential and contribute to society but also abandon its prejudices toward the older adult and integrate them back into the wider community and help them integrate into society. For example, Japan’s digital inclusion initiatives in public libraries have ensured that digitally disadvantaged older adult people can better enjoy library services. And then, by involving governments, markets, and universities, promoting education for the older adult can break down invisible barriers to social participation and digital obstacles. France’s experience in this regard is worth learning from, as the French government has incorporated older adult education into the national education system and provided planning direction and financial support to ensure its success. Finally, communities should actively encourage and promote volunteer services, experience forums, and other activities that enable the older adult to “remain productive,” supporting them in contributing their remaining energy.

Thirdly, we should fully leverage our geographical advantages. In the eastern region, we encourage science and technology enterprises and research institutes to develop intelligent products and services for the older adult, such as wearable devices and telemedicine. Furthermore, we should promote the integration of the senior care service industry with tourism, culture, education and other sectors to create new economic growth points. Meanwhile, the eastern regions offer a relatively abundant array of entertainment and cultural activities. While catering to the needs of young people, these companies can also add operational modules for the older adult to meet the entertainment and social needs of trendy seniors who pursue a higher quality of life. In the central region, a diversified senior care service system should be established to cater to different needs. This includes home care, community care, and institutional care. Leveraging the strategy of the rise of central China, social capital investment in the senior care industry should be encouraged through favorable policies. For instance, the German government exempts property taxes on land used for the older adult care industry and sets a minimum land price ceiling to ensure the supply of land for this purpose. In France, tax incentives are provided through preferential tax rates and tax exemptions on value-added tax to encourage the provision of older adult care services. The western region should take advantage of its rich natural resources to develop ecological and tourism-based retirement care. Unique ethnic cultures can be utilized to organize cultural activities and festivals. Additionally, infrastructure development, health management, medical services, and the social security system should be improved.

Fourthly, regional linkages should be realized. Each region must recognize its own strengths and weaknesses in politics, economy, culture, environment and other aspects. These strengths should be distilled into empirical knowledge. And then share with national urban agglomerations in a timely manner to achieve cross-regional development. For example, Yangpu District in Shanghai has been deeply exploring and utilizing its territorial resources. Based on its status as a national model city for national fitness, it has innovatively created a “sports+” community sports and health center through multi-department collaboration, which deeply integrates national fitness and national health. This center prioritizes the older adult as its key service targets, providing them with sports and wellness services such as health monitoring, fitness guidance, knowledge dissemination, leisure, and social activities. The center has now matured and formed a model that is being promoted in many provinces and cities in China. At the same time, neighboring regions should collaborate to build development clusters for active aging based on their unique resources and characteristics. This collaboration will form a spatial pattern of complementary advantages. To further enrich the linkage of non-neighboring regions, the National Office on Aging should establish an information interaction platform. This platform would showcase the work on aging in different regions, share model cases and experiences, and provide a window for national exchange and cooperation.

## Data Availability

Publicly available datasets were analyzed in this study. This data can be found here: https://charls.pku.edu.cn.

## Footnotes

^1^ https://charls.pku.edu.cn
